# A multi-event combination maintenance model based on event correlation

**DOI:** 10.1371/journal.pone.0207390

**Published:** 2018-11-26

**Authors:** Chunhui Guo, Chuan Lyu, Jiayu Chen, Dong Zhou

**Affiliations:** 1 School of Reliability and Systems Engineering, Beihang University, Beijing, China; 2 State Key Laboratory of Virtual Reality Technology and Systems, Beihang University, Beijing, China; 3 Science and Technology on Reliability and Environmental Engineering Laboratory, Beihang University, Beijing, China; Nanjing University of Information Science and Technology, CHINA

## Abstract

Due to the complexity of large production systems, maintenance events are diverse, simultaneous and dynamic. Appropriate maintenance management of complex large production systems can guarantee high availability and save maintenance costs. However, current maintenance decision-making methods mainly focus on the maintenance events of single-components and series connection multi-components; little research pays attention to the combination maintenance of different maintenance events. Therefore, this paper proposes a multi-event combination maintenance model based on event correlation. First, the maintenance downtime and cost of three types of maintenance events under different maintenance beginning times and degrees are analysed. Then, shared maintenance downtime and cost models are established by maintenance event correlations. In addition, a multi-event combination maintenance model is constructed to achieve the goal of the highest availability and the lowest cost rate in both the decision-making cycle and the remaining life. Moreover, a particle swarm optimization algorithm based on interval segmentation for model solving is designed. Finally, a numerical example is presented to illustrate the model.

## 1. Introduction

Maintenance cost of modern production systems occupies a large proportion of the entire cost cycle [1–3]. Therefore, the importance of maintenance management is also gradually highlighted. If there is no reasonable maintenance decision, not only will it waste maintenance labour and cost but also it consumes a certain amount of maintenance resources. At the same time, it may produce downtime cost and reduce the effective use time of the production system. Therefore, it is necessary and urgent to formulate reasonable and effective maintenance strategies. Complex large production system has a wide range of coexisting maintenance events. In addition, maintenance events are dynamically updated due to the constant operation of production. Therefore, maintenance events are diverse, simultaneous and dynamic. The proper and effective management of these maintenance events is crucial.

There is much research in this field. At present, the research of maintenance decision modelling is mainly divided into two parts, single-component and multi-component methods. The maintenance decision-making model for a single-component occurs more frequently, and the method is mature. The study of multi-components is mostly assumed as a single tandem system. However, little research exists on multi-component systems with complex structure.

For a single-component system, there are five relatively mature maintenance decision-making models including the delay time model, the proportional hazard model, the shock model, the LEVY process model and the Markov decision process model. For the delay time model (DTM), it[4] has been widely applied to the modelling and optimization of inspection of the two-stage failure process for single-component with single failure mode[5–9]. For the proportional hazard model, many attempts have been made to relate the failure probability to both historical service life time and condition monitoring variables[10,11]. For the shock model, it has been successfully applied to many subjects, such as physics, communication, electronic engineering and medicine. As a result, a greater number of researchers have become interested in this topic [12–17]. For the LEVY process model and the Markov decision process model, the LEVY process model is used to solve the problem of determining condition based maintenance policies [18–20]. Single-component maintenance decision models are relatively mature, so it is very effective for the single-component maintenance management problem. However, when a multi-component model is working in a complex large production system, it is inadequate. Therefore, many experts and scholars also conducted in-depth research on multi-component maintenance decision models.

For multi-component systems, there are three relatively mature maintenance decision-making methods including group maintenance, bulk maintenance and opportunity maintenance. For group maintenance, dynamic programming models are presented for determining optimal policies for two and three component equipment [21]. R Dekkert et al. developed a methodology to represent the cost-effectiveness of combining activities and to identify an optimal combination plan [22]. For bulk maintenance, D Assaf et al. considered optimum group maintenance policies for a set of N machines subjected to stochastic failures under continuous and periodic inspections [23]. For opportunity maintenance, RE Wildeman et al. proposed a rolling-horizon approach that takes a long-term tentative plan as the basis for subsequent adaptation according to information that becomes available for the short term [24].

The multi-component maintenance decision model has some shortcomings in the management of complex large production system maintenance events. Most studies focus on multi-component maintenance decisions by assuming that equipment is a whole component or series of connected components [25,26]. However, the actual equipment is a mixed combination of complex production systems including many maintenance events. In addition, most research assumes that the repair degree of the system is to repair to the pre-fault state or repair to an intact state. However, the actual repair process is incompletely repaired. Moreover, there is less consideration for fault retention.

Therefore, this paper proposes a multi-event combination maintenance model based on event correlation for these deficiencies. The model is of great significance to solve the maintenance management of complex large production systems. Combined with the current system health monitoring technology [27,28], real-time decision-making is realized, which can greatly reduce maintenance cost and increase the availability of complex large systems [29–32].

The structure of this paper is organized as follows. Section 2 presents the related work. Section 3 describes the methodology, including the maintenance downtime cost model, the shared maintenance downtime and cost model, a multi-event combination maintenance model and particle swarm optimization algorithm. Section 4 uses a numerical example to verify the accuracy of the model. Finally, the conclusion and discussion are presented in Section 5.

## 2. Related work

To construct the model mentioned in this paper, the following related works are necessary.

1)Degraded Event opportunity maintenance thresholds

Degraded Event refers to the components that degrade during work [5,33,34]. In the degradation process, the opportunity maintenance threshold will be set.

**Fig 1:**
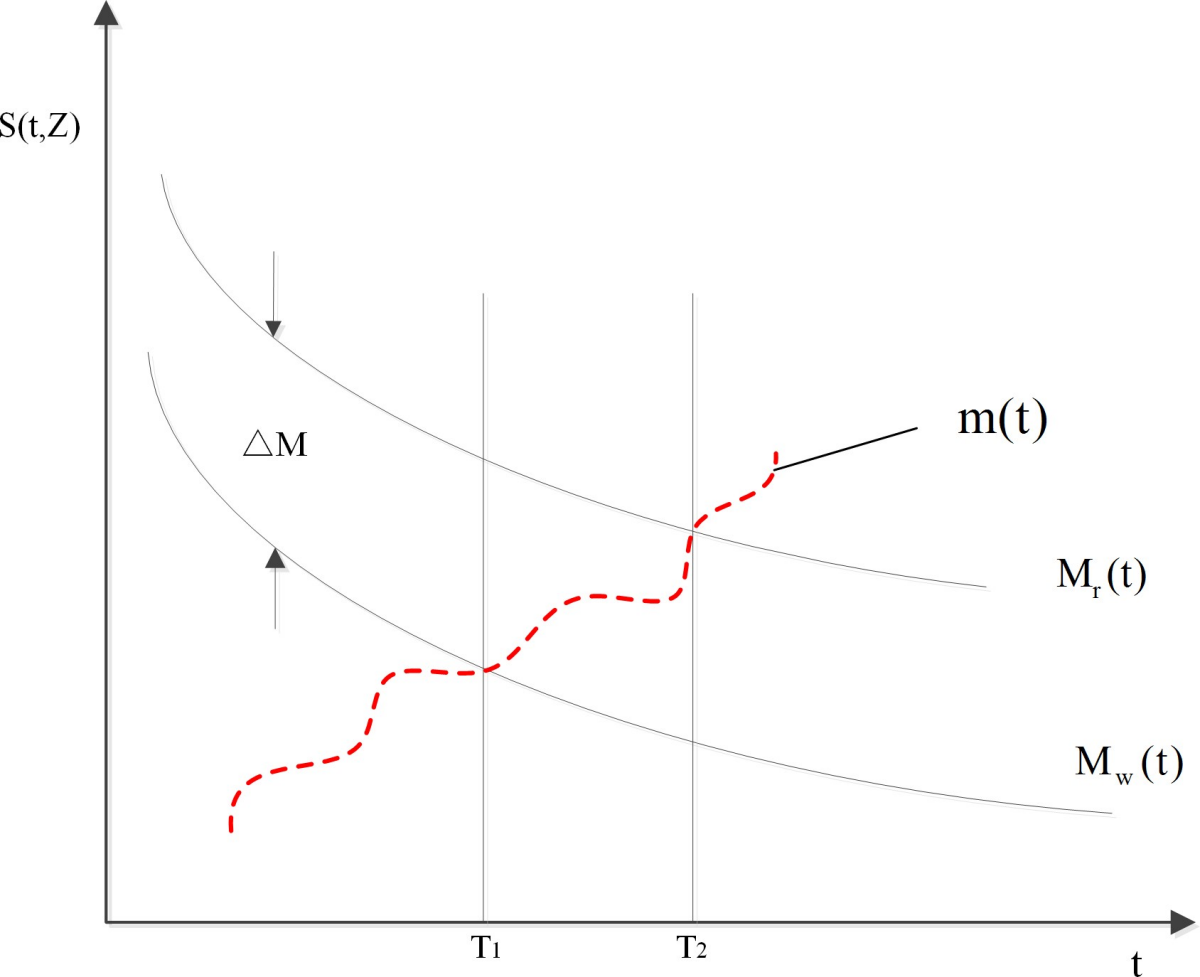
Degradation Event opportunity maintenance thresholds.

Just as Fig 1 shows, the state change during the degradation of components. *m*(*t*), represents the component state. *M*_*r*_(*t*) represents the risk threshold. When the state of the component reaches this level, the component must be repaired. *M*_w_(*t*) is the pre-warning threshold. When the state of the component reaches this level, component degradation begins. S(t,Z) represents the state change function of the component over time. *M*_*r*_(*t*) and *M*_w_(*t*) are changeable over time. ΔM is opportunity maintenance interval. *T*_1_ is the pre-warning threshold time. *T*_2_ is the risk threshold time. [*T*_1_,*T*_2_] is the possible time interval for opportunity maintenance.

2)Timed Event opportunity maintenance thresholds

**Fig 2:**
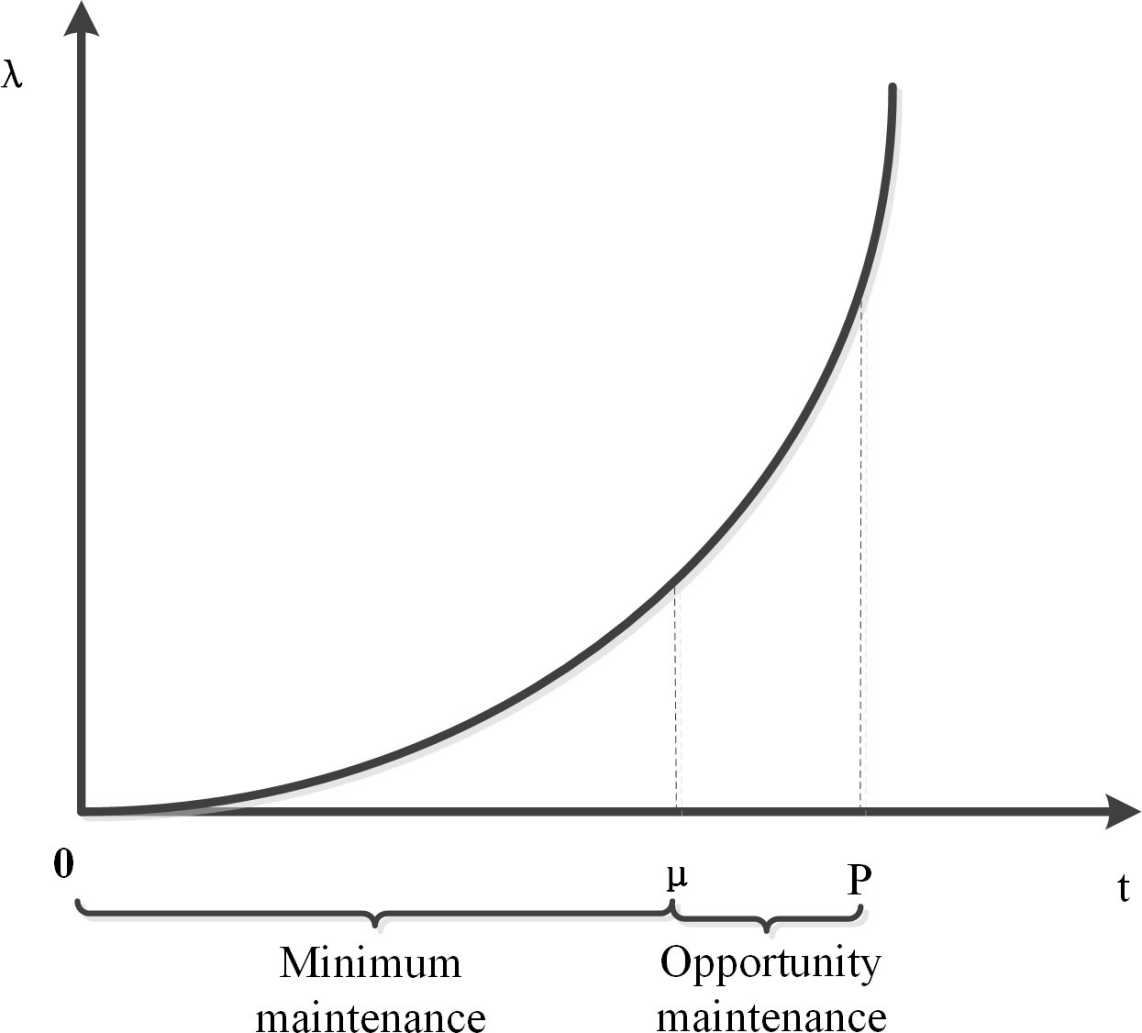
Timed Event opportunity maintenance thresholds.

Just as Fig 2 shows, the Timed Event opportunity maintenance threshold is set as (*μ*,*p*). The detailed derivation process can be referred to [35]. *μ* represents the maximum lead time of the Timed Event. P is the specified repair time for the Timed Event. *λ* represents status of the components. The opportunity maintenance strategies are taken as follows:

- *t*∈[0,*μ*): Minimum maintenance is conducted if a minor fault is detected and complete maintenance is conducted if a major fault is detected.
- *t*∈[*μ*,*P*): Complete maintenance is conducted when minor or major faults occur. If the component does not fail at this time, and other maintenance events are detected in the system, then the component and fault component are repaired together.
- *t* = *P*: This is the specified last repair time.

3) Particle swarm algorithm
  - The particle swarm algorithm is a traditional optimization algorithm [36]. The basic steps are as follows:
  - Initialize the particle swarm: set the population size, and randomly generate each particle position and speed.
  - Construct a fitness function to calculate the fitness of each particle.
  - According to the fitness function value, the optimal position Gbest of all the particles and the best position Pbest [i] of each particle are obtained.
  - Update speed and location of each particle.
  - Finally, by constantly updating, obtain the optimal solution.

Considering the ability of particle swarm optimization to search quickly, when we construct a multi-objective optimization maintenance model, we can make some improvements to the algorithm to solve our model. There is a detailed algorithm design process in 3.4.

## 3. Methodology

A multi-event combination maintenance model based on event correlation in this paper proposes to dissolve the management problem of maintenance events in complex large systems. For complex large-scale systems, maintenance events can be divided into Fault Event, Degradation Event and Timed Event. Fault Event refers to when components fault occur, they need to be repaired afterwards. For Fault Event, it can be classified into Retentive Fault Event and Non-retentive Fault Event, depending on whether fault can be retained. The condition of fault retention is determined by the impact of the fault itself. Degradation Event refers to the degradation of the component performance and it requires preventive maintenance. Timed Event refers to specified maintenance events due to technical requirements or management system regulations. Therefore, this paper focuses on analysing these three types of maintenance events. Due to the two main quantitative maintenance indicators of complex large systems, maintenance time and cost, this paper uses the availability and maintenance cost rates as decision-making goals.

**Fig 3:**
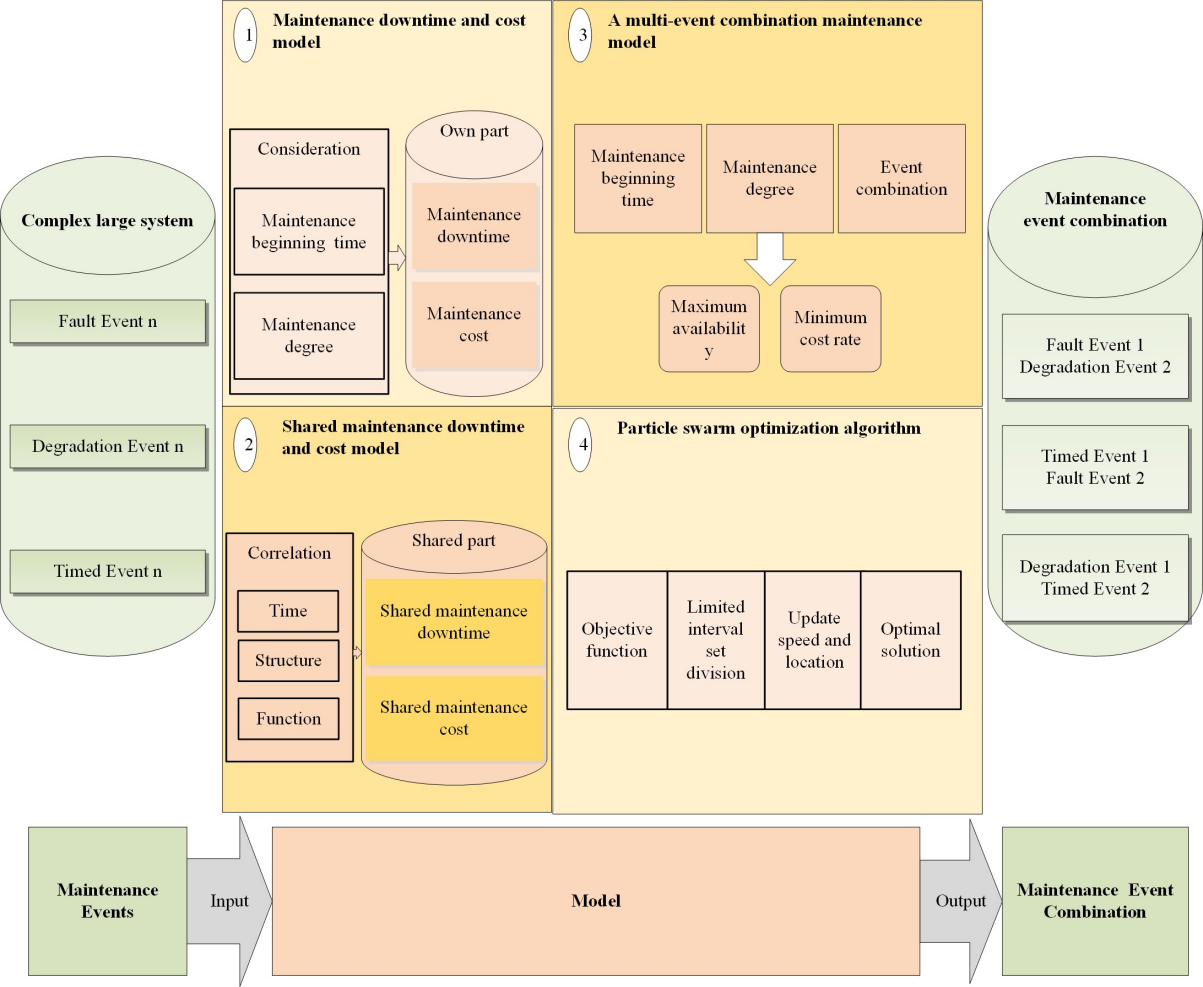
Structure of model construction.

The process of model construction is shown in Fig 3. Model construction includes the following four parts: the maintenance downtime and cost model, the shared maintenance downtime and cost model, a multi-event combination maintenance model and a particle swarm optimization algorithm. The following subsections will be introduced in turn.

### 3.1. Maintenance downtime and cost model

Due to the continuous operation of the complex large system, the state of the components continuously changes over time. Maintenance downtime and cost are affected by component status. Therefore, it is possible to establish a functional relationship between maintenance downtime as well as cost and maintenance beginning time. In addition, different maintenance degrees have different effects on maintenance downtime and cost.

According to the analysis of related work (Timed Event opportunity maintenance threshold), in the Timed Event opportunity maintenance threshold, since the maintenance time and work contents have been determined in advance, maintenance cost C_*pi*_ and maintenance downtime *T*_*pi*_ are assumed constant. Thus, maintenance downtime and the cost model of Fault Event and Degradation Event are the research focus of this paper.

#### 3.1.1. Fault event

1) Non-retentive Fault Event

It is assumed that Non-retentive Fault Event is detected on component *i*, so the maintenance downtime and cost model of component *i* can be expressed as

$$T_{fnri}(t_{i})=T_{\min}(T_{fi})+\delta_{i}(T_{\max}(T_{fi})-T_{\min}(T_{fi}))+H_{1}*(t_{i}-T_{fi})$$ (1)

$$C_{fnri}(t_{i})=C_{\min}(T_{fi})+\delta_{i}(C_{\max}(T_{fi})-C_{\min}(T_{fi}))+H_{2}*(t_{i}-T_{fi})$$ (2)

*T*_*fi*_: The time when a Non-retentive Fault Event is detected.

*t*_*i*_: The maintenance beginning time.

*T*_*fnri*_(*t*_*i*_): The maintenance downtime when Non-retentive Fault Event *i* is handled at the maintenance time *t*_*i*_.

*T*_min_(*T*_*fi*_): The minimum maintenance downtime when a Non-retentive Fault Event is detected at *T*_*fi*_

*T*_max_(*T*_*fi*_): The maximum maintenance downtime when a Non-retentive Fault Event is detected at *T*_*fi*_

*C*_*fnri*_(*t*_*i*_): The maintenance cost when Non-retentive Fault Event *i* is handled at the maintenance time *t*_*i*_.

*C*_min_(*T*_*fi*_): The minimum maintenance cost when a Non-retentive Fault Event is detected at *T*_*fi*_.

*C*_max_(*T*_*fi*_): The maximum maintenance cost when a Non-retentive Fault Event is detected at *T*_*fi*_.

*δ*_*i*_: Maintenance degree. The range is (0,1). *δ*_*i*_ = 0 means minimum maintenance. *δ*_*i*_ = 1 means complete maintenance. 0<*δ*_*i*_<1 means incomplete maintenance.

*H*_1_: Huge maintenance downtime. This means that the Non-retentive Fault Event can only be repaired immediately; otherwise, the maintenance downtime can be long.

*H*_2_: Huge maintenance cost. This means that the Non-retentive Fault Event can only be repaired immediately; otherwise, the maintenance cost cannot be afforded.

2) Retentive Fault Event

It is assumed that a Retentive Fault Event is detected on component *i* in the opportunity maintenance threshold, and the maintenance downtime and cost model of component *i* can be expressed as

$$T_{fri}(t_{i})=T_{\min}(t_{i}-T_{fi})+\delta_{i}(T_{\max}(t_{i}-T_{fi})-T_{\min}(t_{i}-T_{fi}))$$ (3)

$$C_{fri}(t_{i})=C_{\min}(t_{i}-T_{fi})+\delta_{i}(C_{\max}(t_{i}-T_{fi})-C_{\min}(t_{i}-T_{fi}))$$ (4)

*T*_*fi*_: The time when a Retentive Fault Event is detected.

*t*_*i*_: The maintenance beginning time.

*T*_*fri*_(*t*_*i*_): The maintenance downtime when Retentive Fault Event *i* is handled at the maintenance time *t*_*i*_.

*T*_min_(*t*_*i*_−*T*_*fi*_): The minimum maintenance downtime when a Retentive Fault Event is detected at *T*_*fi*_ and is handled at *t*_*i*_.

*T*_max_(*t*_*i*_−*T*_*fi*_): The maximum maintenance downtime when a Retentive Fault Event is detected at *T*_*fi*_ and is handled at *t*_*i*_.

*C*_*fri*_(*t*_*i*_): The maintenance cost when a Retentive Fault Event *i* is handled at the maintenance time *t*_*i*_.

C_min_(*t*_*i*_−*T*_*fi*_): The minimum maintenance cost when a Retentive Fault Event is detected at *T*_*fi*_ and is handled at *t*_*i*_.

C_max_(*t*_*i*_−*T*_*fi*_): The maximum maintenance cost when a Retentive Fault Event is detected at *T*_*fi*_ and is handled at *t*_*i*_.

*δ*_*i*_: Maintenance degree. The range is (0,1). *δ*_*i*_ = 0 means minimum maintenance. *δ*_*i*_ = 1 means complete maintenance. 0<*δ*_*i*_<1 means incomplete maintenance.

3) Fault Event maintenance downtime and cost model construction

The Non-retentive Fault Event and Retentive Fault Event maintenance downtime models can be combined together. The Fault Event maintenance downtime and cost model can be expressed as

$$T_{fi}(t_{i},\delta_{i})=(1-\omega_{fi})*T_{fnri}(t_{i})+\omega_{fi}*T_{fi}(t_{i})$$ (5)

$$C_{fi}=(1-\omega_{fi})*C_{fnri}(t_{i})+\omega_{fi}*C_{fi}(t_{i})$$ (6)

$$\omega_{fi}=\{\begin{array}{c} 0\left(\begin{array}{ccc} \mathrm{Non}‑\mathrm{retentive} & \mathrm{Fault} & \mathrm{Event} \end{array}\right)\\ 1\left(\begin{array}{ccc} \mathrm{Retentive} & \mathrm{Fault} & \mathrm{Event} \end{array}\right) \end{array}$$

#### 3.1.2. Degradation event

According to the analysis of related work (Degraded Event opportunity maintenance threshold) for the Degraded Event opportunity maintenance threshold, the maintenance downtime and cost model of a Degradation Event can be expressed as follows:

$$T_{di}(t_{i},\delta_{i})=T_{\min}(t_{i}-T_{di})+\delta_{i}(T_{\max}(t_{i}-T_{di})-T_{\min}(t_{i}-T_{di}))$$ (7)

$$C_{di}(t_{i},\delta_{i})=C_{\min}(t_{i}-T_{di})+\delta_{i}(C_{\max}(t_{i}-T_{di})-C_{\min}(t_{i}-T_{di}))$$ (8)

*T*_*di*_: The time when a Degradation Event is detected.

*t*_*i*_: The maintenance beginning time.

*T*_*di*_(*t*_*i*_,*δ*_*i*_): The maintenance downtime when Degradation Event *i* is handled at the maintenance time *t*_*i*_.

*T*_min_(*t*_*i*_−*T*_*di*_): The minimum maintenance downtime when a Degradation Event is detected at *T*_*di*_ and is handled at *t*_*i*_.

*T*_max_(*t*_*i*_−*T*_*di*_): The maximum maintenance downtime when a Degradation Event is detected at *T*_*di*_ and is handled at *t*_*i*_.

*C*_*di*_(*t*_*i*_,*δ*_*i*_): The maintenance cost when Degradation Fault Event *i* is handled at the maintenance time *t*_*i*_.

C_min_(*t*_*i*_−*T*_*di*_): The minimum maintenance cost when a Degradation Fault Event is detected at *T*_*di*_ and is handled at *t*_*i*_.

C_max_(*t*_*i*_−*T*_*di*_): The maximum maintenance cost when a Degradation Fault Event is detected at *T*_*di*_ and is handled at *t*_*i*_.

*δ*_*i*_: Maintenance degree. The range is (0,1). *δ*_*i*_ = 0 means minimum maintenance. *δ*_*i*_ = 1 means complete maintenance. 0<*δ*_*i*_<1 means incomplete maintenance.

### 3.2. Shared maintenance downtime and cost model

#### 3.2.1. Maintenance event correlation

To facilitate the combination of the maintenance events, the correlation between maintenance events needs to be analysed. According to engineering experience and expert analysis, at present, maintenance event correlation is generally divided into fault correlation, time correlation, structure correlation and function correlation. The specific meaning of each correlation is shown in Table 1.

**Table 1:** Four correlation specific meaning.

Correlation	Specific meaning
Fault correlation	Fault correlation can be divided into three categories: The first correlation means that when a component fails, other component will be affected to fail by different chance. The second correlation means that when a component fails, failure rate of other related component will be affected. The third correlation means that when a component fails, related components will be impacted. If the impact reaches a certain extent, related component will fails.
Time correlation	A components need repaired, the other components in the system need to be repaired at the same or near time.
Structure correlation	Structure correlation means that there is a certain overlap in maintenance process between two components. In other words, there are the same maintenance steps during the maintenance process of both components.
Function correlation	Function correlation can be divided into two categories: Function correlation Ⅰrefers to that components can share the same maintenance resources because of similar functions; Function correlation II means that shared parts can be repaired when there are multiple fault components in the system.

**Fig 4:**
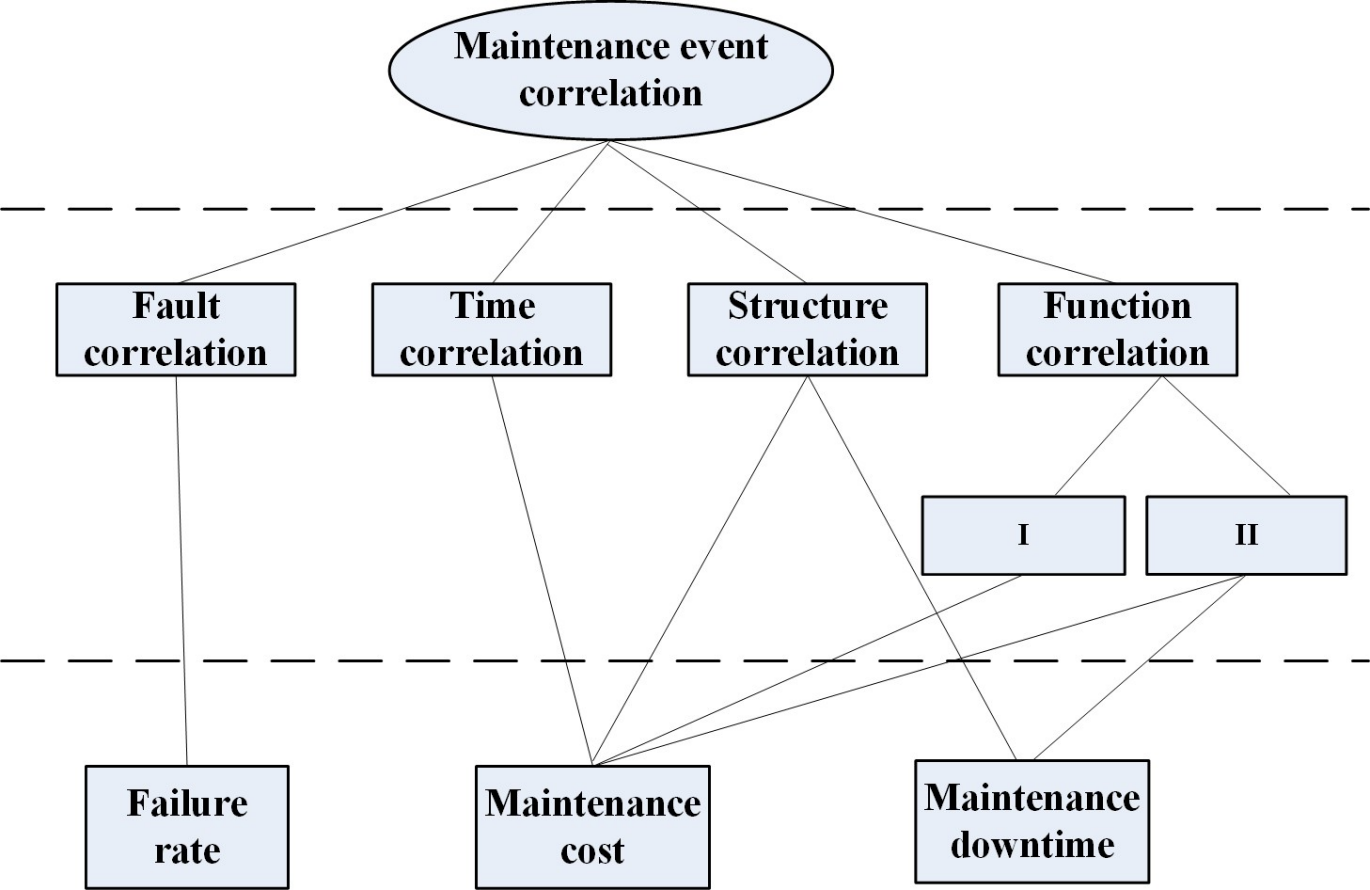
Impact of maintenance event correlation on maintenance downtime and cost.

The impact of maintenance event correlation is shown in Fig 4. Through the analysis of four correlations, fault correlation will have a certain impact on the system failure rate and affect the overall health of the system. Due to time correlation, the maintenance cost will reduce. Due to structure correlation, it is possible to reduce the operation of the overlapping portion, saving maintenance cost and downtime. Due to function correlation I, maintenance costs will be reduced for shared maintenance resources. Due to function correlation II, maintenance costs and maintenance downtime will be saved.

**Fig 5:**
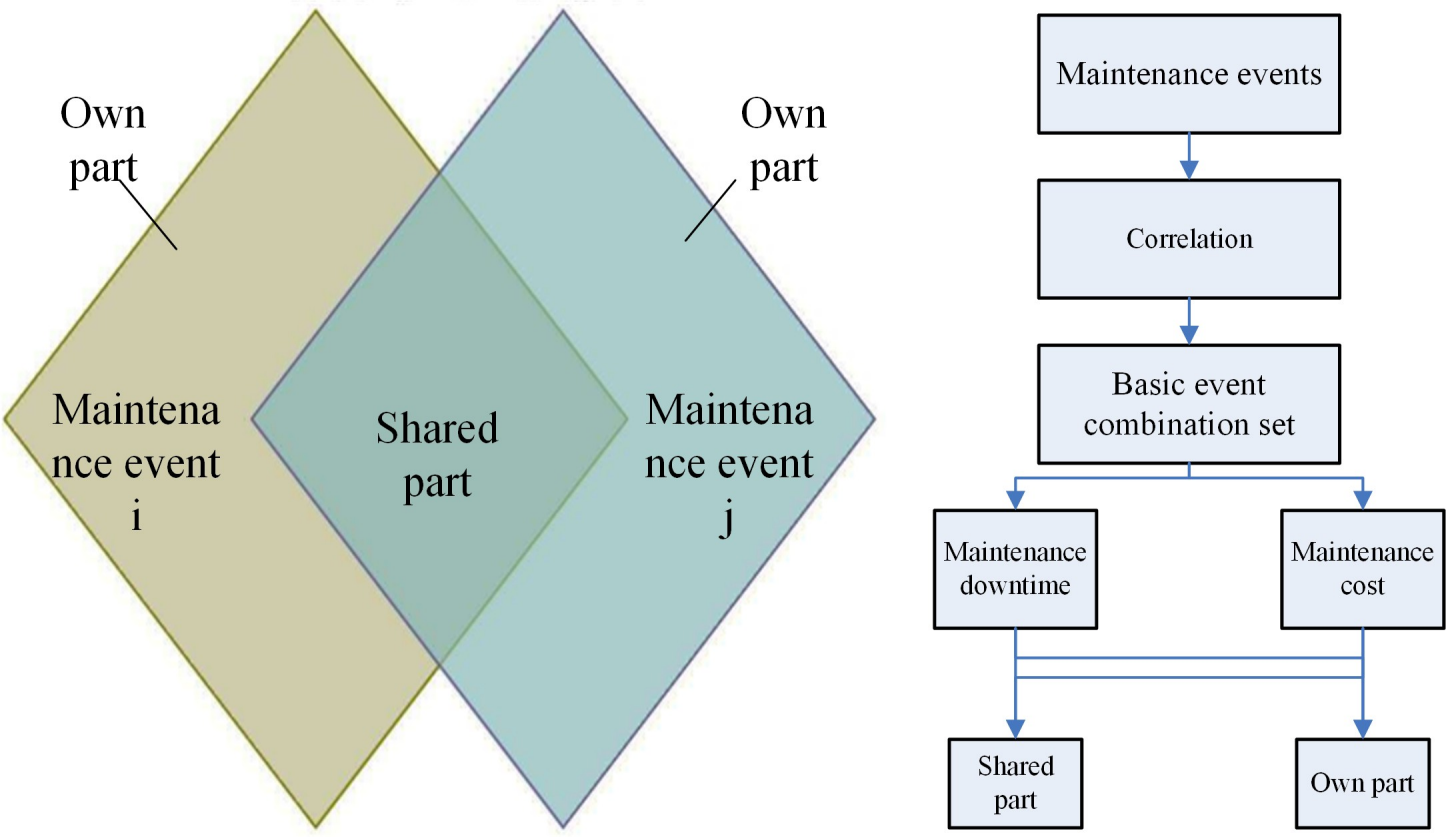
Own part and shared part analysis.

The shared part is generated when the maintenance event is combined, and the remainder is the own part, as shown in Fig 5.

By analysing the characteristics of the event combination, the basic event combination set is established. The own part and the shared part are obtained for the maintenance event combination according to the correlation, laying the foundation for the establishment of a multi-event combination maintenance model.

#### 3.2.2. Shared maintenance downtime model

According to correlation analysis, because of the existence of structure correlation and functional correlation II, the maintenance event combination will reduce maintenance downtime. The following assumptions and notations are made:

- There are *N*_0_(*t*) components needing repair at time t.
- $\Delta T_{ij}^{1}(t)$: shared downtime at time t due to the structural correlation between component i and j.
- $\Delta T_{ij}^{2}(t)$: shared downtime at time t due to functional correlation II between component i and j.

The shared maintenance downtime model construction process is as follows:

Matrix B_*i*_: the correlation between maintenance event i and other maintenance events

$$\begin{array}{l} \begin{array}{cc} \;1\;2\;\ldots & j\;\ldots \;N_{0} \end{array}\\ B_{i}=\begin{array}{c} \mathrm{Structure}\;\mathrm{correlation}\\ \mathrm{Function}\;\mathrm{correaltion}\;\mathrm{II} \end{array}\left[\begin{array}{l} \begin{array}{cccccc} b_{i1}^{1} & b_{i2}^{1} & \ldots & b_{ij}^{1} & \ldots & b_{in}^{1} \end{array}\\ \begin{array}{cccccc} b_{i1}^{2} & b_{i2}^{2} & \ldots & b_{ij}^{2} & \ldots & b_{in}^{2} \end{array} \end{array}\right] \end{array}$$ (9)

$$b_{ij}^{1},\;b_{ij}^{2}=\{\begin{array}{c} 0\;(\mathrm{no}\;\mathrm{correlation})\\ 1(\mathrm{existing}\;\mathrm{correlation}) \end{array}$$

$T_{2\times N_{0}}^{i}$: Shared maintenance downtime between maintenance event i and other maintenance events according to the two correlations.

$$T_{2\times N_{0}}^{i}=\left[\begin{array}{ccccc} \Delta T_{i1}^{1} & \Delta T_{i2}^{1} & \ldots & \Delta T_{i(N_{0}-1)}^{1} & \Delta T_{iN_{0}}^{1}\\ \Delta T_{i1}^{2} & \Delta T_{i2}^{2} & \cdots & \Delta T_{i(N_{0}-1)}^{2} & \Delta T_{iN_{0}}^{2} \end{array}\right]$$ (10)

Therefore, the shared maintenance downtime model can be expressed as

$$S_{2\times N_{0}}^{i}=\left[\begin{array}{ccccc} b_{i1}^{1}*\Delta T_{i1}^{1} & b_{i2}^{1}*\Delta T_{i2}^{1} & \ldots & b_{i(N_{0}-1)}^{1}*\Delta T_{i(N_{0}-1)}^{1} & b_{iN_{0}}^{1}*\Delta T_{iN_{0}}^{1}\\ b_{i1}^{2}*\Delta T_{i1}^{2} & b_{i2}^{2}*\Delta T_{i2}^{2} & \cdots & b_{i(N_{0}-1)}^{2}*\Delta T_{i(N_{0}-1)}^{2} & b_{iN_{0}}^{2}*\Delta T_{iN_{0}}^{2} \end{array}\right]$$ (11)

Shared maintenance total downtime between maintenance event i and other maintenance events due to two correlations can be expressed as below.

$$T^{i}=\sum_{k=1}^{2}\sum_{j=1}^{N_{0}}s_{ij}^{k}$$ (12)

$$s_{ij}^{k}=b_{ij}^{k}*\Delta T_{ij}^{k},\;k=1,2,\;j=1,2,3\ldots \ldots N_{0}$$

The correlation of two maintenance events is mutual, so according to the above method, all maintenance events shared maintenance downtime is calculated twice. Thus, the shared maintenance downtime for all the maintenance events needs to be halved.

$$\Delta T(t)=\frac{1}{2}*\sum_{i=1}^{N_{0}}\sum_{k=1}^{2}\sum_{j=1}^{N_{0}}s_{ij}^{k}$$ (13)

#### 3.2.3. Shared maintenance cost model

According to correlation analysis, because of the existence of time correlation, structural correlation and functional correlation, the maintenance event combination will reduce maintenance costs. The following assumptions and notations are made:

- There are *N*_0_(*t*) components needing repair at time t.
- *C*_*Stop*_(*t*): Downtime loss of unit time when a maintenance event is conducted at t.
- *C*_*Fixed*_: Fixed shared maintenance cost.
- *T*_*i*_(*t*): Maintenance beginning time of maintenance event i.
- *T*_*j*_(*t*): Maintenance beginning time of maintenance event j.
- wi={1,Maintenanceeventsineeddowntimemaintenance0,Maintenanceeventsidon'tneeddowntimemaintenance
- wj={1,Maintenanceeventsjneeddowntimemaintenance0,Maintenanceeventsjdon'tneeddowntimemaintenance
  1) Shared maintenance cost according to time correlation

Shared maintenance cost according to the time correlation between maintenance event i and maintenance event j can be expressed as

$$△C_{ij}^{1}=\left\{ \begin{array}{l} C_{Fixed}^{ij}(t)+w_{i}*w_{j}*T_{i}(t)*C_{Stop}(t)\begin{array}{cc} & T_{i}(t)\leq T_{j}(t); \end{array}\\ C_{Fixed}^{ij}(t)+w_{i}*w_{j}*T_{j}(t)*C_{Stop}(t)\begin{array}{cc} & T_{j}(t)\leq T_{i}(t); \end{array} \end{array}\right.$$ (14)

2) Shared maintenance cost according to functional correlation

Maintenance cost can be shared for shared maintenance resources (function correlation i) and for reducing logistics delay times (function correlation ii). Therefore, shared maintenance cost according to functional correlation can be expressed as

$$△C_{ij}^{2}=C_{Fixed}^{ij}(t)+w_{i}*w_{j}*C_{stop}(t)*\min\left\{T_{i}(t),T_{j}(t)\right\}+\Delta T_{ij}^{2}(t)*C_{stop}(t)$$ (15)

3) Shared maintenance cost according to structural correlation

Shared maintenance cost according to structural correlation between maintenance event I and maintenance event j can be expressed as

$$\Delta C_{ij}^{3}=C_{{}_{Fixed}}^{ij}+w_{i}*w_{j}*C_{stop}(t)*\min\left\{T_{i}(t),T_{j}(t)\right\}+C_{p}*\Delta T_{ij}^{1}(t)$$ (16)

$$C_{p}:\;\mathrm{Unit}\;\mathrm{labour}\;\mathrm{cost}.$$

4) Shared maintenance cost model

Matrix *A*_*i*_: The correlation between maintenance event i and other maintenance events.

$$\begin{array}{l} \;\begin{array}{c} 1\;2\;\ldots \;j\;\ldots \;N_{0} \end{array}\\ A_{i}=\begin{array}{c} \mathrm{Time}\;\mathrm{correlation}\\ \mathrm{Function}\;\mathrm{correlation}\\ \mathrm{Structure}\;\mathrm{correlation} \end{array}\left[\begin{array}{l} \begin{array}{cccccc} a_{i1}^{1} & a_{i2}^{1} & \ldots & a_{ij}^{1} & \ldots & a_{iN_{0}}^{1} \end{array}\\ \begin{array}{cccccc} a_{i1}^{2} & a_{i2}^{2} & \ldots & a_{ij}^{2} & \ldots & a_{iN_{0}}^{2} \end{array}\\ \begin{array}{cccccc} a_{i1}^{3} & a_{i2}^{3} & \ldots & a_{ij}^{3} & \ldots & a_{iN_{0}}^{3} \end{array} \end{array}\right] \end{array}$$ (17)

$$a_{ij}^{1},\;a_{ij}^{2},\;a_{ij}^{3}=\{\begin{array}{c} 0(no\;correlation)\\ 1(existing\;correlation) \end{array}$$

$C_{3\times N_{0}}^{i}$ represents the shared maintenance cost between maintenance event i and other maintenance events according to the three correlations

$$C_{3\times N_{0}}^{i}=\left[\begin{array}{ccccc} \Delta C_{i1}^{1} & \Delta C_{i2}^{1} & \ldots & \Delta C_{i(N_{0}-1)}^{1} & \Delta C_{iN_{0}}^{1}\\ \Delta C_{i1}^{2} & \Delta C_{i2}^{2} & \cdots & \Delta C_{i(N_{0}-1)}^{2} & \Delta C_{iN_{0}}^{2}\\ \Delta C_{i1}^{3} & \Delta C_{i2}^{3} & \cdots & \Delta C_{i(N_{0}-1)}^{3} & \Delta C_{iN_{0}}^{3} \end{array}\right]$$ (18)

Therefore, the shared maintenance cost model can be expressed as

$$D_{3\times N_{0}}^{i}=\left[\begin{array}{ccccc} a_{i1}^{1}*\Delta C_{i1}^{1} & a_{i2}^{1}*\Delta C_{i2}^{1} & \ldots & a_{i(N_{0}-1)}^{1}*\Delta C_{i(N_{0}-1)}^{1} & a_{iN_{0}}^{1}*\Delta C_{iN_{0}}^{1}\\ a_{i1}^{2}*\Delta C_{i1}^{2} & a_{i2}^{2}*\Delta C_{i2}^{2} & \cdots & a_{i(N_{0}-1)}^{2}*\Delta C_{i(N_{0}-1)}^{2} & a_{iN_{0}}^{2}*\Delta C_{iN_{0}}^{2}\\ a_{i1}^{3}*\Delta C_{i1}^{3} & a_{i2}^{3}*\Delta C_{i2}^{3} & \cdots & a_{i(N_{0}-1)}^{3}*\Delta C_{i(N_{0}-1)}^{3} & a_{iN_{0}}^{3}*\Delta C_{iN_{0}}^{3} \end{array}\right]$$ (19)

The shared maintenance total cost between maintenance event i and other maintenance events due to the three correlations can be expressed as

$$C_{i}=\sum_{k=1}^{3}\sum_{j=1}^{N_{0}}d_{ij}^{k}‑\sum_{j=1}^{N_{0}}\left(\sum_{k=1}^{3}a_{ij}^{k}-1)(C_{Fixed}^{ij}+C_{stop}(t)*\min\left\{T_{i}(t),T_{j}(t)\right\}\right)$$ (20)

$$d_{ij}^{k}=a_{ij}^{k}*\Delta C_{ij}^{k}\;k=1,2,3,\;j=1,2,3\ldots \ldots n.$$

The correlation of two maintenance events is mutual; according to the above method all maintenance events shared maintenance cost is calculated twice, so the shared maintenance cost for all the maintenance events needs to be halved.

$$\Delta C=\frac{1}{2}*\sum_{i=1}^{N_{0}}C_{i}$$ (21)

### 3.3. A multi-event combination maintenance model

#### 3.3.1. Notation and assumptions

- There are n components in the system numbered from 1 to n in order.
- (*λ*_di_, *λ*_fi_): The failure rate threshold of opportunity maintenance for component i.
- (T_di_, T_fi_): The degradation threshold of opportunity maintenance for component i.
- (T_pi_, T_hi_): The timed threshold of opportunity maintenance for component i.
- t_*s*_: Decision-making start time.
- t_*i*_: Repair time of maintenance event i.
- T_0_: Decision-making cycle.
- N_0_: The total number of maintenance events in decision-making cycle T_0_.
- n_1_: The number of Failure Events in decision-making cycle T_0_.
- n_2_:The number of Degradation Events in decision-making cycle T_0_.
- n_3_: The number of Timed Events in decision-making cycle T_0_.
- C_*fi*_(*t*): Maintenance cost function of a Retention Fault Event.
- C_*di*_(*t*): Maintenance cost function of a Degradation Event.
- C_*pi*_: Maintenance cost function of a Timed Event.
- $R_{i}=\frac{1}{i!}\sum_{k=0}^{i-1}{\left(-1\right)}^{k}\left[\begin{array}{l} i\\ k \end{array}\right]{\left(i-k\right)}^{N_{0}}$: Maintenance event combination number. *N*_0_ maintenance events are arbitrarily divided into i blocks.
- 1~^n_1_^: the serial number of the Failure Event.
- ^n_1_^+1~^n_1_^+^n_2_^: The serial number of the Degradation Event.
- ^n_1_^+^n_2_^~^n_1_^+^n_2_^+^n_3_^:The number of the Timed Event.
- *C*_*fmi*_: Replacement cost of component i.
- $T_{work}^{i}$: Fault-free working hours of component I before t_*i*_.
- *f*_*i*_(*t*|*Z*(*t*)): Failure rate density function of component i.
- *R*_*L*_(*t*): the system reliability function at time t in the remaining life cycle.
- *T*_*ml*_: required maintenance downtime once.

#### 3.3.2. Model construction

Suppose there are three dummy variables, *ω*_*fi*_, *ω*_*di*_, and *ω*_p*i*_.

$$\omega_{fi}=\{\begin{array}{l} 0\;\left(\begin{array}{ccc} \mathrm{Non}‑\mathrm{retentive} & \mathrm{Fault} & \mathrm{Event} \end{array}\right)\\ 1\;\left(\begin{array}{ccc} \mathrm{Retentive} & \mathrm{Fault} & \mathrm{Event} \end{array}\right) \end{array}$$ (22)

$$\omega_{di}=\left\{ \begin{array}{l} 0\begin{array}{cc} , & m_{i}(t)<M_{w}^{i}(t) \end{array}\\ 1\begin{array}{cc} , & M_{w}^{i}(t)\leq m_{i}(t) \end{array} \end{array}\right.$$ (23)

$$\omega_{pi}=\left\{ \begin{array}{l} 0\begin{array}{cc} , & t_{i}<T_{\mathrm{pi}} \end{array}\\ 1\begin{array}{cc} , & t_{i}\geq T_{\mathrm{pi}} \end{array} \end{array}\right.$$ (24)

Thus, $n_{1}=\sum_{i=1}^{n}\omega_{fi}$, $n_{2}=\sum_{i=1}^{n}\omega_{di}$, $n_{3}=\sum_{i=1}^{n}\omega_{pi}$, N_0_ = n_1_+n_2_+n_3_

The combination matrix when maintenance events are arbitrarily divided into i blocks is as follows:

$$B_{R_{i}\times i}=\left[\begin{array}{ccc} B_{11} & \ldots \ldots & B_{1i}\\ B_{21} & \ldots \ldots & B_{2i}\\ \ldots \ldots & \ldots \ldots & \ldots \ldots \\ B_{R_{i}1} & \ldots \ldots & B_{R_{i}i} \end{array}\right]$$ (25)

The kth sub-combination of the jth combination:

$$B_{jk}=\left[\begin{array}{ccc} b_{{}_{j1}}^{k} & \ldots \ldots & b_{jr_{jk}}^{k} \end{array}\right]$$ (26)

*r*_*jk*_: Maintenance event number in B_*jk*_.

$$\sum_{k=1}^{i}r_{jk}=N_{0}$$ (27)

$b_{{}_{jr_{jk}}}^{k}$: The serial number of maintenance events.

Shared maintenance cost matrix:

$$\Delta C_{R_{i}\times i}=\left[\begin{array}{ccc} \Delta c_{11} & \ldots \ldots & \Delta c_{1i}\\ \Delta c_{22} & \ldots \ldots & \Delta c_{2i}\\ \ldots \ldots & \ldots \ldots & \ldots \ldots \\ \Delta c_{R_{i}1} & \ldots \ldots & \Delta c_{R_{i}i} \end{array}\right]$$ (28)

Δc_*jk*_: The shared maintenance cost of the kth sub-combination of the jth combination when *N*_0_ maintenance events are arbitrarily divided into i blocks

$$\Delta c_{jk}=\frac{1}{2}*\sum_{n=1}^{{}_{r_{jk}}}C_{n}$$ (29)

The shared maintenance cost of all the maintenance events is expressed as follows:

$$\Delta C_{ij}=\sum_{k=1}^{i}\Delta c_{jk}=\frac{1}{2}*\sum_{k=1}^{i}\sum_{n=1}^{{}_{r_{jk}}}C_{n}$$ (30)

The maintenance cost after combination maintenance can be expressed as

$$C_{ij}(t_{1},\;t_{2}\ldots t_{N_{0}},\;\delta_{1},\;\delta_{2}\ldots \delta_{N_{0}})=\sum_{i=1}^{n_{1}}C_{fi}(t_{i},T_{fi},\delta_{i})+\sum_{i=n_{1}+1}^{n_{1}+n_{2}}C_{di}(t_{i},T_{di},\delta_{i})+\sum_{i=n_{1}+n_{2}+1}^{N_{0}}C_{pi}-\Delta C_{ij}$$ (31)

Complete maintenance restores components to the initial condition; the minimum maintenance restores the failure rate back to the moment before the fault. Incomplete maintenance makes components return to a state before repair.

The maintenance cost in the remaining life period considering the impact of repair degree on the failure rate and the number of maintenance of remaining life cycle can be expressed as

$$C^{'}=\sum_{i=1}^{n}\left(\int_{t_{s}+T_{0}}^{\infty }f_{i}((t-\eta_{i}*\delta_{i}*T_{work}^{i})|Z(t))dt)\right)*C_{fmi}$$ (32)

$$\eta_{i}=\left\{ \begin{array}{l} 1,\;\mathrm{maintenance}\\ 0,\;\mathrm{no}\;\mathrm{maintenance} \end{array}\right.$$

The mean value of the remaining life cycle of the system is

$$T_{rl}=\int_{0}^{\infty }R_{L}(t)dt=\int_{0}^{\infty }f(f_{1}(t|Z(t)),\cdots f_{i}(t|Z(t)),\cdots f_{n}(t|Z(t)))dt$$ (33)

$$f_{i}(t|Z(t))=f_{i}((t+T_{i}-\delta_{i}*T_{i})|Z(t))$$ (34)

The maintenance cost rate after combination maintenance can be expressed as

$$S=\frac{C_{i}(t_{1},\;t_{2}\ldots t_{N_{0}},\;\delta_{1},\;\delta_{2}\ldots \delta_{N_{0}})+C^{'}}{T_{0}+T_{rl}}$$ (35)

The shared maintenance downtime matrix is

$$\Delta T_{R_{i}\times i}=\left[\begin{array}{ccc} \Delta t_{11} & \ldots \ldots & \Delta t_{1i}\\ \Delta t_{22} & \ldots \ldots & \Delta t_{2i}\\ \ldots \ldots & \ldots \ldots & \ldots \ldots \\ \Delta t_{R_{i}1} & \ldots \ldots & \Delta t_{R_{i}i} \end{array}\right]$$ (36)

Δ*t*_*jk*_: The shared maintenance downtime of the kth sub-combination of the jth combination when *N*_0_ maintenance events are arbitrarily divided into i blocks.

The shared maintenance downtime of all the maintenance events is expressed as follows:

$$\Delta T=\frac{1}{2}*\sum_{j=1}^{R_{i}}\Delta t_{j}$$ (37)

$$\Delta t_{j}=\frac{1}{2}*\sum_{k=1}^{i}\Delta t_{jk}$$ (38)

The maintenance downtime in the remaining life period considering the impact of repair degree on the failure rate and the number of maintenance of remaining life cycle can be expressed as

$$T^{'}=\sum_{i=1}^{n}\left(\int_{t_{s}+T_{0}}^{\infty }f_{i}((t-\eta_{i}*\delta_{i}*T_{work}^{i})|Z(t))dt)\right)*T_{ml}$$ (39)

$$\eta_{i}=\left\{ \begin{array}{l} 1\;,\;\mathrm{maintenance}\\ 0\;,\;\mathrm{no}\;\mathrm{maintenance} \end{array}\right.$$

Availability after combination maintenance can be expressed as

$$A_{0}=\frac{T_{0}+T_{rl}-(\sum_{i=1}^{n_{1}}T_{fi}(t_{i},T_{fi},\delta_{i})+\sum_{i=n_{1}+1}^{n_{1}+n_{2}}T_{di}(t_{i},T_{di},\delta_{i})+\sum_{i=n_{1}+n_{2}+1}^{N_{0}}T_{pi}-\Delta T)-T^{'}}{T_{0}+T_{rl}}$$ (40)

A multi-event combination maintenance model is expressed as follows to achieve the goal of the highest availability and the lowest cost rate in not only the decision-making cycle but also the remaining life:

$$\left\{ \begin{array}{l} \min\;S(t_{1},\;t_{2}\ldots t_{n},\;\delta_{1},\;\delta_{2}\ldots \delta_{n})\\ \max\;A(t_{1},\;t_{2}\ldots t_{n},\;\delta_{1},\;\delta_{2}\ldots \delta_{n})\\ s.t.\;t_{s}\leq t_{i}\leq T_{i}(0<i\leq n_{1})\\ s.t.\;t_{s}\leq t_{i}\leq T_{fi}(n_{1}<i\leq n_{1}+n_{2})\\ s.t.\;t_{s}\leq t_{i}\leq T_{hi}(n_{1}+n_{2}<i\leq N_{0})\\ s.t.\;\sum_{i=1}^{n}\omega_{fi}<N \end{array}\right.$$ (41)

### 3.4. Particle swarm optimization algorithm

**Fig 6:**
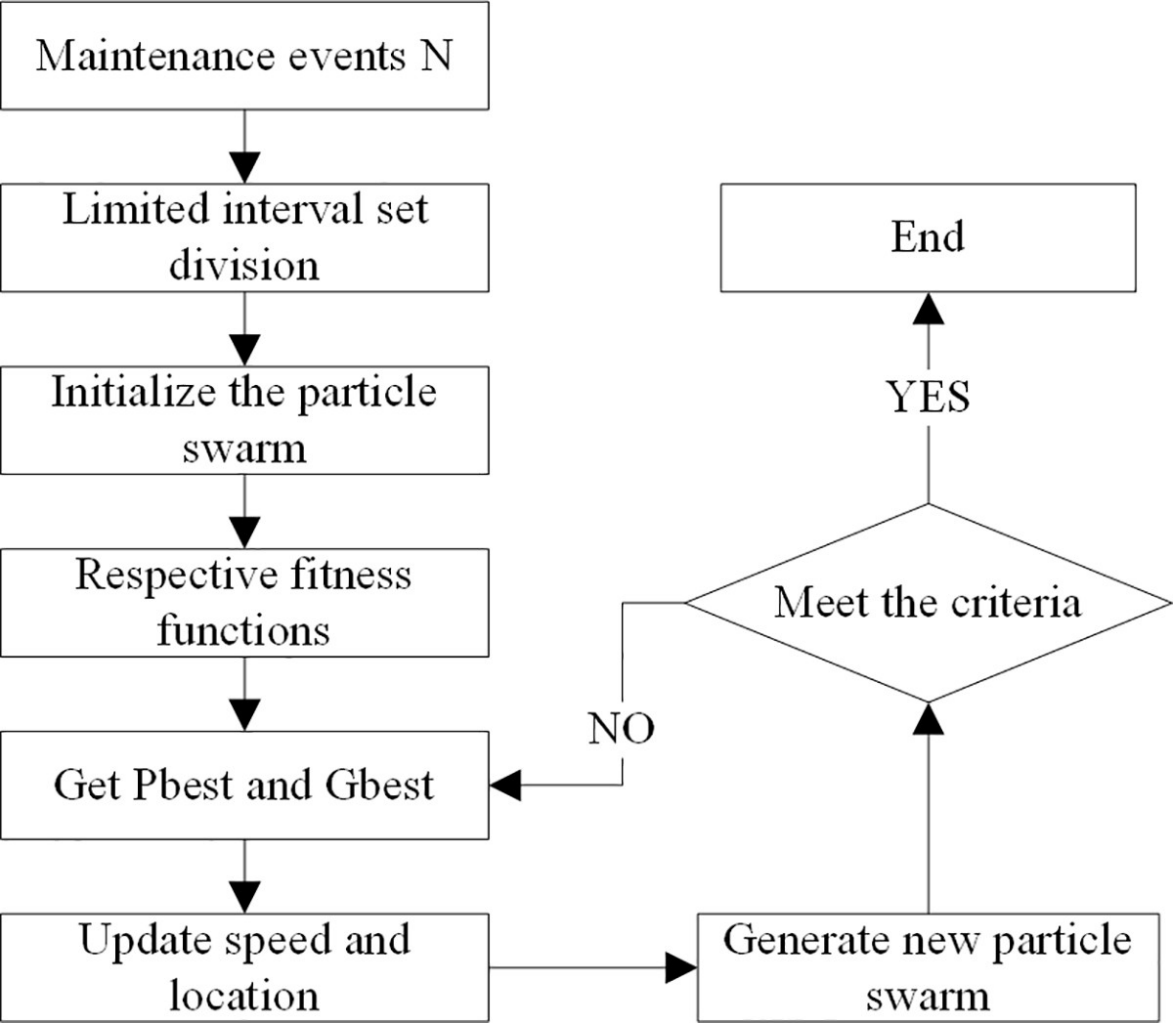
A particle swarm optimization algorithm flow based on interval segmentation.

According to the model features, a particle swarm optimization algorithm based on interval segmentation is designed. Algorithm flow is as shown in Fig 6.

#### 3.4.1. Interval segmentation

- According to the health status and maintenance plan of the system, the number of different types of events is counted as N.
- Obtain event set A = {1,2, …, i, … N};
- The set A is divided into separate $\sum_{i=1}^{N}\frac{1}{i!}\sum_{k=0}^{i-1}{\left(-1\right)}^{k}\left[\begin{array}{l} i\\ k \end{array}\right]{\left(i-k\right)}^{N}$ sub-combinations, where the number of i separate sub-combinations is $\frac{1}{i!}\sum_{k=0}^{i-1}{\left(-1\right)}^{k}\left[\begin{array}{l} i\\ k \end{array}\right]{\left(i-k\right)}^{N}$.

#### 3.4.2. Multi-objective particle swarm optimization

Two objective functions can be expressed in vector form $\min\;y=f(t_{1},t_{2}\ldots t_{N})=(S(t_{1},t_{2}\ldots t_{N}),\bar{A}(t_{1},t_{2}\ldots t_{N}))\;\bar{A}(t_{1},t_{2}\ldots t_{N})$: Availability function.

*S*(*t*_1_,*t*_2_…*t*_*N*_): Maintenance cost rate function.

- Initialize the particle swarm: set the population size to 50.
- According to the characteristics of the objective function, to achieve two goals of the lowest maintenance cost rate and the maximum availability, the function of the maintenance cost rate and the availability function are calculated as the respective fitness functions.
  $$Fitness[i,1]=S(t_{1},t_{2}\ldots t_{N})$$
  $$Fitness[i,2]=\bar{A}(t_{1},t_{2}\ldots t_{N})$$
- According to 2.3, the particle swarm algorithm, the optimal solution is obtained.

## 4. Numerical example

In this numerical example, we examine the model developed earlier and assess the validity of its development. From the model notation, a large number of opportunity maintenance intervals, maintenance costs and downtime are carefully considered. In particular, the relationship between some of the cost and downtime parameters must be reasonably specified. We start with some basic model parameters, which need careful consideration. There are 4 components in the system, and the component numbers are 1, 2, 3, and 4. The system life is 50,000 hours. The last trouble-free working hours of components 1, 2, 3, and 4 were 400, 500, 600, and 300 hours in system records, respectively. t_*s*_ = 1000 is the decision-making start time. T_0_ = 50 is the decision-making cycle. During the decision-making cycle (1000, 1050), four maintenance events have been detected in the system. The Retention Fault Event number is 1, the Non-retentive Fault Event number is 2, the Degraded Event number is 3, and the Timed Event number is 4; the maintenance event number and component number are the same. As is shown from the system data, there is a structural correlation between component 1 and component 2. There is a functional correlation between component 3 and component 4, and there is a time correlation between component 1 and component 4. The four maintenance events corresponding to the opportunity maintenance thresholds are (990,1040), (1000,1020), (980,103), and (1010,1045). The unit downtime cost is 1000 / hour. Components 1, 2, and 4 are consistent with the exponential distribution, and the failure rate function is 0.01. Component 3 conforms to the Weibull distribution with a failure rate function of $\frac{2.651}{4294.112}{\left(\frac{t}{4294.112}\right)}^{1.651}$ We assume the maintenance cost and downtime as follows:

- The minimum maintenance cost function for component 1 is $C_{\min}^{1}(t)=\frac{1}{8}t+100$. The complete maintenance cost function is $C_{\max}^{1}(t)=\frac{1}{2}t^{2}+200$. The corresponding maintenance cost function with the retention time is
  $$C_{f1}(t)=\frac{1}{8}t+100+\delta_{1}*(\frac{1}{2}t^{2}-\frac{1}{8}t+100).$$
- The minimum maintenance cost for component 2 is 100, and the complete maintenance cost is 300; the corresponding maintenance cost function with retention time is
  $$C_{f2}(t)=100+200*\delta_{2}+10000000*t.$$
- The minimum maintenance cost for component 3 with degradation time is $C_{\min}^{3}(t)=\frac{1}{9}t+100$. The complete maintenance cost is $C_{\max}^{3}(t)=\frac{1}{9}t^{2}+200$. The corresponding maintenance cost function with the degradation time is
  $$C_{d3}(t)=\frac{1}{9}t+100+\delta_{3}*\left[\frac{1}{9}(t^{2}-t)+100\right].$$
- The maintenance cost function of Timed Event 4 due to preventive maintenance in advance is C_*p*4_(*t*) = 30.
- The minimum maintenance downtime for component 1 with retention time is $T_{\min}^{1}(t)=\frac{1}{100}t+0.2$. The complete maintenance downtime is $T_{\max}^{1}(t)=\frac{1}{50}t+0.4$. The corresponding maintenance downtime for the retention time is
  $$T_{f1}(t)=(1+\delta_{1})*(\frac{1}{100}t+0.2).$$
- The minimum maintenance downtime of component 2 is 0.2 and the complete maintenance downtime is 0.5; the corresponding maintenance downtime with retention time is
  $$T_{f2}(t)=10000000*t+0.2+0.3*\delta_{2}.$$
- The minimum maintenance downtime of Degradation Event 3 is $T_{\min}^{3}(t)=\frac{1}{50}t+0.1$. The complete maintenance downtime is $T_{\max}^{3}(t)=\frac{1}{25}t+0.2$. The corresponding maintenance downtime for the degradation time is $T_{di}(t)=(\frac{1}{50}t+0.1)*(1+\delta_{3})$.
- Timed Event 4 maintenance downtime is 0.15, and the repair degree is 0.8.
- The shared maintenance cost function for component 1 and component 2 due to structure correlation with retention time is
  $$\Delta C_{12}^{3}(t)=80+\delta_{1}*\delta_{2}*t+1000*\min(T_{f1}(t),T_{f2}(t)).$$
- The shared maintenance cost function for components 3 and 4 due to function correlation with degradation time is
  $$\Delta C_{34}^{2}(t)=90+0.1*\delta_{3}*t+1000*\min(T_{di}(t),0.15)+0.1*\delta_{3}*t$$
- The shared maintenance cost for component 1 and 4 due to time correlation is min(*T*_*f*1_(*t*),0.15)*1000.
- The shared maintenance downtime function for component 1 and component 2 due to structural correlation with the retention time is
  $$\Delta T_{12}=1.5*\min(T_{f1}(t),T_{f2}(t)).$$
- The shared maintenance downtime for component 3 and component 4 due to functional correlation is 0.1+min(*T*_*d*3_(*t*),0.15).
- The shared maintenance downtime for component 1 and component 4 due to time correlation is min(*T*_*f*1_(*t*),0.15).

According to the multi-event combination maintenance model and its algorithm, 50 particles are selected and iteratively run 100 times; the optimization results are obtained. By controlling the maintenance cost and time when the failure of 3 components occurred in the remaining life cycle, the results listed in Table 2, Table 3 and Table 4 can be obtained.

**Table 2:** Optimization results analysis when follow-up maintenance cost and downtime is small.

Number	Combination	Cost rate	Availability	Maintenance beginning time	Maintenance degree
1	{1,2,3,4}	25005.3381	-24999.01075	*t*_1_ = *t*_2_ = *t*_3_ = *t*_4_ = 1010	*δ*_1_ = 0,*δ*_2_ = 0,*δ*_3_ = 0
2	{1,3,4} {2}	5.477152894	0.989520534	*t*_1_ = *t*_3_ = *t*_4_ = 1015, *t*_2_ = 1000	*δ*_1_ = 0,*δ*_2_ = 0,*δ*_3_ = 0
3	{1,3} {2,4}	25005.57617	-24999.01027	*t*_1_ = *t*_3_ = 1000, *t*_2_ = *t*_4_ = 1010	*δ*_1_ = 0,*δ*_2_ = 0,*δ*_3_ = 0
4	{1,4} {2,3}	5.563570771	0.989678261	*t*_1_ = *t*_4_ = 1030, *t*_2_ = *t*_3_ = 1000	*δ*_1_ = 0,*δ*_2_ = 0,*δ*_3_ = 0
5	{1} {2,3,4}	25005.56732	-24999.01036	*t*_1_ = 1020, *t*_2_ = *t*_3_ = *t*_4_ = 1010	*δ*_1_ = 0,*δ*_2_ = 0,*δ*_3_ = 0
6	{1,2,4} {3}	25005.53404	-24999.01033	*t*_1_ = *t*_2_ = *t*_4_ = 1010, *t*_3_ = 1000	*δ*_1_ = 0,*δ*_2_ = 0,*δ*_3_ = 0
7	{1,2} {3,4}	5.445757596	0.989460319	*t*_1_ = *t*_2_ = 1000, *t*_3_ = *t*_4_ = 1015	*δ*_1_ = 0,*δ*_2_ = 0,*δ*_3_ = 0
8	{1,2,3} {4}	5.572856055	0.989739149	*t*_1_ = *t*_2_ = *t*_3_ = 1000, *t*_4_ = 1045	*δ*_1_ = 0,*δ*_2_ = 0,*δ*_3_ = 0
9	{1,4} {2} {3}	5.525206885	0.989595935	*t*_1_ = *t*_4_ = 1030, *t*_2_ = 1000, *t*_3_ = 1000	*δ*_1_ = 0,*δ*_2_ = 0,*δ*_3_ = 0
10	{1}{2,4} {3}	25005.56198	-24999.01032	*t*_1_ = 1000, *t*_2_ = *t*_4_ = 1010, *t*_3_ = 1000	*δ*_1_ = 0,*δ*_2_ = 0,*δ*_3_ = 0
11	{1} {2} {3,4}	5.531188743	0.989578401	*t*_1_ = 1000, *t*_2_ = 1000, *t*_3_ = *t*_4_ = 1012	*δ*_1_ = 0,*δ*_2_ = 0,*δ*_3_ = 0
12	{1,3} {2} {4}	5.568581195	0.989673718	*t*_1_ = *t*_3_ = 1010, *t*_2_ = 1000, *t*_4_ = 1045	*δ*_1_ = 0,*δ*_2_ = 0,*δ*_3_ = 0
13	{1} {2,3} {4}	5.567349491	0.989718591	*t*_1_ = 1011, *t*_2_ = *t*_3_ = 1000, *t*_4_ = 1045	*δ*_1_ = 0,*δ*_2_ = 0,*δ*_3_ = 0
14	{1,2} {3} {4}	5.498783943	0.989627319	*t*_1_ = *t*_2_ = 1000, *t*_3_ = 1000, *t*_4_ = 1045	*δ*_1_ = 0,*δ*_2_ = 0,*δ*_3_ = 0
15	{1} {2} {3} {4}	5.501215063	0.989642937	*t*_1_ = 1000, *t*_2_ = 1000, *t*_3_ = 1000, *t*_4_ = 1045	*δ*_1_ = 0,*δ*_2_ = 0,*δ*_3_ = 0

**Table 3:** Optimization results analysis when follow-up maintenance cost and downtime take the middle value.

Number	Combination	Cost rate	Availability	Maintenance beginning time	Maintenance degree
1	{1,2,3,4}	25005.49479	-24999.01116	*t*_1_ = *t*_2_ = *t*_3_ = *t*_4_ = 1010	*δ*_1_ = 0,*δ*_2_ = 0,*δ*_3_ = 0
2	{1,3,4} {2}	5.621700391	0.989041404	*t*_1_ = *t*_3_ = *t*_4_ = 1020, *t*_2_ = 1000	*δ*_1_ = 0, *δ*_2_ = 0, *δ*_3_ = 0
3	{1,3} {2,4}	25005.73151	-24999.01076	*t*_1_ = *t*_3_ = 1000, *t*_2_ = *t*_4_ = 1010	*δ*_1_ = 0, *δ*_2_ = 0, *δ*_3_ = 10.134192425
4	{1,4} {2,3}	5.709435166	0.989242843	*t*_1_ = *t*_4_ = 1023, *t*_2_ = *t*_3_ = 1000	*δ*_1_ = 0,*δ*_2_ = 0, *δ*_3_ = 0.898110702
5	{1} {2,3,4}	25005.71621	-24999.01079	*t*_1_ = 1020, *t*_2_ = *t*_3_ = *t*_4_ = 1010	*δ*_1_ = 0, *δ*_2_ = 0, *δ*_3_ = 0.709168122
6	{1,2,4} {3}	25005.67628	-24999.01077	*t*_1_ = *t*_2_ = *t*_4_ = 1010, *t*_3_ = 1000	*δ*_1_ = 0, *δ*_2_ = 0, *δ*_3_ = 0.713129444
7	{1,2} {3,4}	5.593594905	0.989039895	*t*_1_ = *t*_2_ = 1000, *t*_3_ = *t*_4_ = 1010	*δ*_1_ = 0, *δ*_2_ = 0, *δ*_3_ = 0
8	{1,2,3} {4}	5.715223423	0.989298807	*t*_1_ = *t*_2_ = *t*_3_ = 1000, *t*_4_ = 1045	*δ*_1_ = 0, *δ*_2_ = 0, *δ*_3_ = 0.681909028
9	{1,4} {2} {3}	5.681863896	0.98920751	*t*_1_ = *t*_4_ = 1014, *t*_2_ = 1000, *t*_3_ = 1000	*δ*_1_ = 0, *δ*_2_ = 0, *δ*_3_ = 0.259964239
10	{1}{2,4} {3}	25005.71236	-24999.01077	*t*_1_ = 1023, *t*_2_ = *t*_4_ = 1010, *t*_3_ = 1000	*δ*_1_ = 0, *δ*_2_ = 0, *δ*_3_ = 0
11	{1} {2} {3,4}	5.680029528	0.989145388	*t*_1_ = 1000, *t*_2_ = 1000, *t*_3_ = *t*_4_ = 1018	*δ*_1_ = 0, *δ*_2_ = 0, *δ*_3_ = 0
12	{1,3} {2} {4}	5.714633773	0.989213142	*t*_1_ = *t*_3_ = 1012, *t*_2_ = 1000, *t*_4_ = 1045	*δ*_1_ = 0, *δ*_2_ = 0, *δ*_3_ = 0
13	{1} {2,3} {4}	5.705892768	0.989264074	*t*_1_ = 1020, *t*_2_ = *t*_3_ = 1000, *t*_4_ = 1045	*δ*_1_ = 0, *δ*_2_ = 0, *δ*_3_ = 0.723926514
14	{1,2} {3} {4}	5.64213591	0.989190305	*t*_1_ = *t*_2_ = 1000, *t*_3_ = 1000, *t*_4_ = 1045	*δ*_1_ = 0, *δ*_2_ = 0, *δ*_3_ = 0.449833708
15	{1} {2} {3} {4}	5.701374627	0.989242271	*t*_1_ = 1000, *t*_2_ = 1000, *t*_3_ = 1000, *t*_4_ = 1045	*δ*_1_ = 0, *δ*_2_ = 0, *δ*_3_ = 0.859645459

**Table 4:** Optimization results analysis when follow-up maintenance cost and downtime is large.

Number	Combination	Cost rate	Availability	Maintenance beginning time	Maintenance degree
1	{1,2,3,4}	25058.6269	-24999.01194	*t*_1_ = *t*_2_ = *t*_3_ = *t*_4_ = 1010	*δ*_1_ = 0,*δ*_2_ = 0,*δ*_3_ = 1
2	{1,3,4} {2}	58.76488409	0.988311517	*t*_1_ = *t*_3_ = *t*_4_ = 1010, *t*_2_ = 1000	*δ*_1_ = 0,*δ*_2_ = 0,*δ*_3_ = 1
3	{1,3} {2,4}	25058.85394	-24999.01146	*t*_1_ = *t*_3_ = 1000, *t*_2_ = *t*_4_ = 1010	*δ*_1_ = 0,*δ*_2_ = 0,*δ*_3_ = 1
4	{1,4} {2,3}	58.84421905	0.988494321	*t*_1_ = *t*_4_ = 1025, *t*_2_ = *t*_3_ = 1000	*δ*_1_ = 0,*δ*_2_ = 0,*δ*_3_ = 1
5	{1} {2,3,4}	25058.85169	- 24999.0116	*t*_1_ = 1021, *t*_2_ = *t*_3_ = *t*_4_ = 1010	*δ*_1_ = 0,*δ*_2_ = 0,*δ*_3_ = 1
6	{1,2,4} {3}	25058.81175	-24999.01151	*t*_1_ = *t*_2_ = *t*_4_ = 1010, *t*_3_ = 1000	*δ*_1_ = 0,*δ*_2_ = 0,*δ*_3_ = 1
7	{1,2} {3,4}	58.73156628	0.988250227	*t*_1_ = *t*_2_ = 1000, *t*_3_ = *t*_4_ = 1010	*δ*_1_ = 0,*δ*_2_ = 0,*δ*_3_ = 1
8	{1,2,3} {4}	58.85056738	0.988550767	*t*_1_ = *t*_2_ = *t*_3_ = 1000, *t*_4_ = 1045	*δ*_1_ = 0,*δ*_2_ = 0,*δ*_3_ = 1
9	{1,4} {2} {3}	58.81790724	-0.988473675	*t*_1_ = *t*_4_ = 1010, *t*_2_ = 1000, *t*_3_ = 1000	*δ*_1_ = 0,*δ*_2_ = 0,*δ*_3_ = 1
10	{1}{2,4} {3}	25058.85078	- 24999.01147	*t*_1_ = 1007, *t*_2_ = *t*_4_ = 1010, *t*_3_ = 1000	*δ*_1_ = 0,*δ*_2_ = 0,*δ*_3_ = 1
11	{1} {2} {3,4}	58.81393401	-0.988355128	*t*_1_ = 1000, *t*_2_ = 1000, *t*_3_ = *t*_4_ = 1010	*δ*_1_ = 0,*δ*_2_ = 0,*δ*_3_ = 1
12	{1,3} {2} {4}	58.85056738	0.988550767	*t*_1_ = *t*_3_ = 1000, *t*_2_ = 1000, *t*_4_ = 1045	*δ*_1_ = 0,*δ*_2_ = 0,*δ*_3_ = 1
13	{1} {2,3} {4}	58.84691514	0.988536673	*t*_1_ = 1007, *t*_2_ = *t*_3_ = 1000, *t*_4_ = 1045	*δ*_1_ = 0,*δ*_2_ = 0,*δ*_3_ = 1
14	{1,2} {3} {4}	58.77403989	0.98841632	*t*_1_ = *t*_2_ = 1000, *t*_3_ = 1000, *t*_4_ = 1045	*δ*_1_ = 0,*δ*_2_ = 0,*δ*_3_ = 1
15	{1} {2} {3} {4}	58.7862767	0.988437615	*t*_1_ = 1000, *t*_2_ = 1000, *t*_3_ = 1000, *t*_4_ = 1045	*δ*_1_ = 0,*δ*_2_ = 0,*δ*_3_ = 1

Assuming that the maintenance costs were 200, 250, 200, and 100 and the maintenance times were 0.3, 0.45, 0.4, and 0.25 when the failure of components 1, 2, 3, and 4 occurred in the remaining life cycle, respectively, the results in Table 2 can be obtained.

Assuming that the maintenance costs were 200, 250, 400, and 100 and maintenance times were 0.3, 0.45, 1.2, and 0.25 when the failure of components 1, 2, 3, and 4 occurred in the remaining life cycle, respectively, the results in Table 3 can be obtained.

Assuming that the maintenance costs were 200, 250, 20000, and 100 and maintenance times were 0.3, 0.45, 4, and 0.25 when the failure of components 1, 2, 3, and 4 occurred in the remaining life cycle, respectively, the results in Table 4 can be obtained.

According to the results in Table 2, Table 3 and Table 4, since the range of the objective function is not limited, some combinations have negative availability. These results are contrary to reality and should be removed. Through comprehensive analysis of the optimization results of the three tables, when the maintenance cost and downtime of component 3 are different, different maintenance degrees have different effects. When the follow-up maintenance cost and downtime are small, the minimum maintenance is more economical; when the follow-up maintenance costs and maintenance downtime are median values, it is economical to determine incomplete maintenance downtime; when the follow-up maintenance costs and maintenance downtime are large, complete maintenance is more economical. This is consistent with actual engineering experience. Of course, the most appropriate maintenance beginning time is also extremely important. The maintenance beginning time in the table is the best time to conduct maintenance, while the cost and downtime generated are also the least.

**Fig 7:**
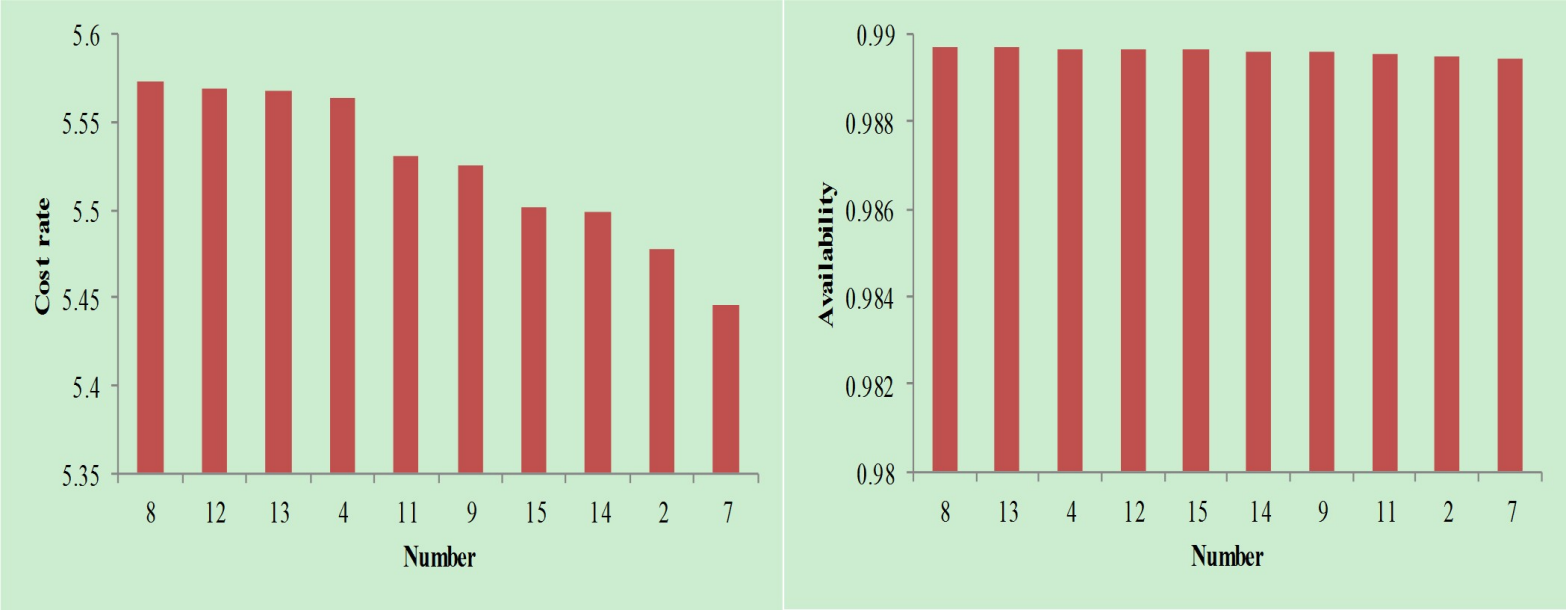
Cost rate and availability analysis when follow-up maintenance cost and downtime are small.

**Fig 8:**
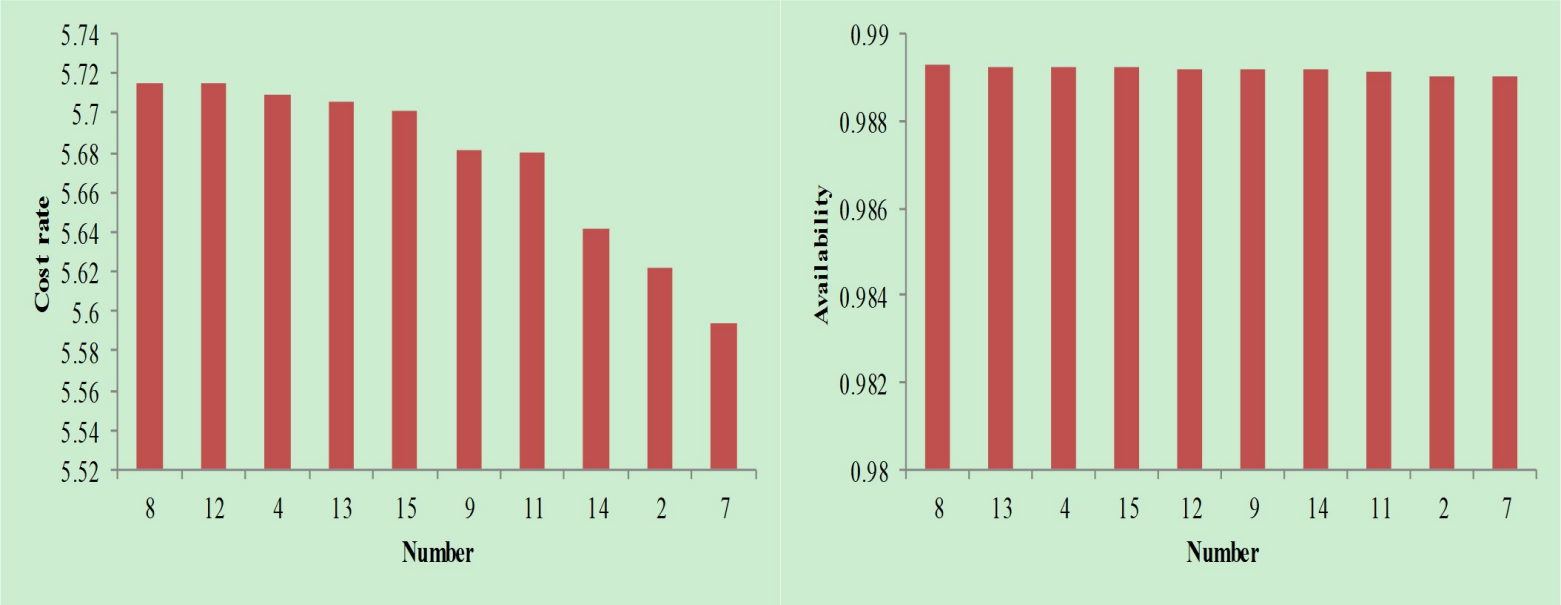
Cost rate and availability analysis when follow-up maintenance cost and downtime take the middle value.

**Fig 9:**
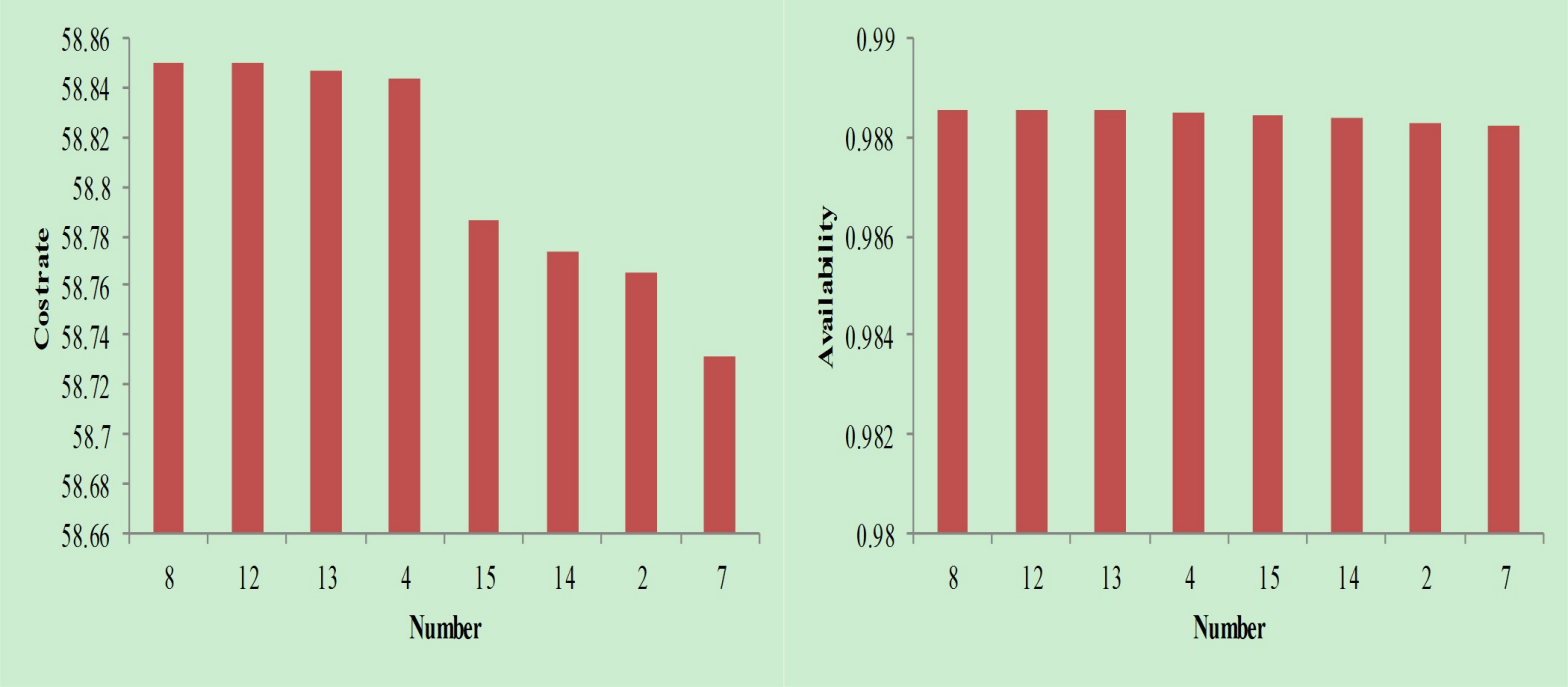
Cost rate and availability analysis when follow-up maintenance cost and downtime are large.

Removing the combination with negative availability in the tables, the results in Fig 7, Fig 8 and Fig 9 can be obtained.

As seen in Fig 7, Fig 8 and Fig 9, the combinations shown are available maintenance combinations. Among them, combination 15 is the traditional maintenance mode. The remaining combination is the optimized combination. From the perspective of availability, combination 8 achieves the highest availability; however, the availability of each combination is relatively close. From the perspective of cost rate, combination 7 achieves the lowest cost rate. Compared with other combinations, the reduction in the cost rate is more obvious. Therefore, through comprehensive consideration of availability and cost rate factors, combination 7 is the best maintenance combination.

Therefore, the decisions at Event 1 and Event 2 are combined to begin maintenance at t = 1000; Event 3 and Event 4 are combined to begin maintenance at t = 1015. Considering the assumption, Event 1 and Event 2 have structural correlation while Event 3 and Event 4 have functional correlation, so the decision-making results meet the assumption, which prove that this decision-making model is accurate.

## 5. Conclusion and discussion

The model method in this paper is used to solve the problem of maintenance event management in complex large-scale production systems. Aiming at the diversity, simultaneity and dynamics of maintenance events, a multi-event combination maintenance model is constructed to achieve the goal of the highest availability and the lowest cost rate in the decision-making cycle and the remaining life of the system. The combination of maintenance events makes maintenance more scientific and standardized.

The new contributions of this paper are as follows:

- The maintenance correlation is summarized into four categories, based on which of the correlations, shared maintenance downtimes and cost models are constructed.
- For traditional single decision-making variables and single decision-making objectives of the maintenance decision-making model method, the maintenance combination of different events, repair times and degrees are considered as optimization variables. The multi-event combination maintenance model is constructed to achieve the goal of the highest availability and the lowest cost rate in the decision-making cycle and the remaining life of the system.

In this paper, we assume that the maintenance cost function and the maintenance downtime function are linear functions of the maintenance degree. However, in actually, maintenance cost and the downtime function have complex function forms. Therefore, in the future, maintenance cost and downtime functions of different equipment under different maintenance beginning times and degrees need more research for accurate decision-making.

## Supporting information

S1 Data (DOCX)
