# Supplementary material for: A multi-event combination maintenance model based on event correlation
Source: PLoS One. 2018 Nov 26;13(11):e0207390. doi: 10.1371/journal.pone.0207390 (PMC6261041; doi:10.1371/journal.pone.0207390)
Supplement: S1 Data — (DOCX) [file pone.0207390.s001.docx]

**Supporting Information**

**S1 Data**

There are 4 components in the system, and the component numbers are 1, 2, 3, and 4. The system life is 50,000 hours. The last trouble-free working hours of components 1, 2, 3, and 4 were 400, 500, 600, and 300 hours in system records, respectively.$\text{t}_{s}\text{=1000}$is the decision-making start time. $\text{T}_{0}\text{=50}$ is the decision-making cycle. During the decision-making cycle (1000, 1050), four maintenance events have been detected in the system. The Retention Fault Event number is 1, the Non-retentive Fault Event number is 2, the Degraded Event number is 3, and the Timed Event number is 4; the maintenance event number and component number are the same. As is shown from the system data, there is a structural correlation between component 1 and component 2. There is a functional correlation between component 3 and component 4, and there is a time correlation between component 1 and component 4. The four maintenance events corresponding to the opportunity maintenance thresholds are (990,1040), (1000,1020), (980,103), and (1010,1045). The unit downtime cost is 1000 / hour. Components 1, 2, and 4 are consistent with the exponential distribution, and the failure rate function is 0.01. Component 3 conforms to the Weibull distribution with a failure rate function of $\frac{2.651}{4294.112}(\frac{t}{4294.112})^{1.651}$

We assume the maintenance cost and downtime as follows:

1. The minimum maintenance cost function for component 1 is$\text{C}_{\min}^{1}(t)\text{=}\frac{1}{8}t+100$. The complete maintenance cost function is $\text{C}_{\max}^{1}(t)\text{=}\frac{1}{2}t^{2}+200$. The corresponding maintenance cost function with the retention time is

$\text{C}_{f1}(t)\text{=}\frac{1}{8}t+100+\delta_{1}*(\frac{1}{2}t^{2}-\frac{1}{8}t+100)$.

1. The minimum maintenance cost for component 2 is 100, and the complete maintenance cost is 300; the corresponding maintenance cost function with retention time is

$\text{C}_{f2}(t)\text{=100+200}*\delta_{2}\text{+}10000000*t$.

1. The minimum maintenance cost for component 3 with degradation time is$\text{C}_{\min}^{3}(t)\text{=}\frac{1}{9}t+100$. The complete maintenance cost is $\text{C}_{\max}^{3}(t)\text{=}\frac{1}{9}t^{2}+200$. The corresponding maintenance cost function with the degradation time is

$\text{C}_{d3}(t)\text{=}\frac{1}{9}t+100\text{+}\delta_{3}*\left[\frac{1}{9}\text{（}t^{2}-t)+100\right]$.

1. The maintenance cost function of Timed Event 4 due to preventive maintenance in advance is $\text{C}_{p4}(t)=30$.
2. The minimum maintenance downtime for component 1 with retention time is $T_{\min}^{1}(t)\text{=}\frac{1}{100}t+0.2$. The complete maintenance downtime is $T_{\max}^{1}(t)\text{=}\frac{1}{50}t+0.4$. The corresponding maintenance downtime for the retention time is

$T_{f1}(t)\text{=}(1+\delta_{1})*(\frac{1}{100}t+0.2)$.

1. The minimum maintenance downtime of component 2 is 0.2 and the complete maintenance downtime is 0.5; the corresponding maintenance downtime with retention time is

$T_{f2}(t)\text{=}10000000*t+0.2+0.3*\delta_{2}$.

1. The minimum maintenance downtime of Degradation Event 3 is$T_{\min}^{3}(t)\text{=}\frac{1}{50}t+0.1$. The complete maintenance downtime is $T_{\max}^{3}(t)\text{=}\frac{1}{25}t+0.2$. The corresponding maintenance downtime for the degradation time is$T_{di}(t)=(\frac{1}{50}t+0.1)*(1+\delta_{3})$.
2. Timed Event 4 maintenance downtime is 0.15, and the repair degree is 0.8.
3. The shared maintenance cost function for component 1 and component 2 due to structure correlation with retention time is

$\Delta C_{12}^{3}(t)=80+\delta_{1}*\delta_{2}*t\text{+}1000\text{*}\min(T_{f1}(t),T_{f2}(t))$.

1. The shared maintenance cost function for components 3 and 4 due to function correlation with degradation time is

$$\Delta C_{34}^{2}(t)=90\text{+}0.1*\delta_{3}*t+1000*\min(T_{di}(t),0.15)+0.1*\delta_{3}*t$$

1. The shared maintenance cost for component 1 and 4 due to time correlation is$\min(T_{f1}(t),0.15)*1000$.
2. The shared maintenance downtime function for component 1 and component 2 due to structural correlation with the retention time is

$\Delta T_{12}=1.5*\min(T_{f1}(t),T_{f2}(t))$.

1. The shared maintenance downtime for component 3 and component 4 due to functional correlation is $\text{0.1+}\min(T_{d3}(t),0.15)$.
2. The shared maintenance downtime for component 1 and component 4 due to time correlation is$\min(T_{f1}(t),0.15)$.
